# Lower range of serum uric acid level increases risk of rapid decline of kidney function in young and middle-aged adults: the Yuport Medical Checkup Center Study

**DOI:** 10.1007/s10157-023-02318-0

**Published:** 2023-02-11

**Authors:** Hitomi Ueda, Kazuo Inoue, Reiko Seki, Yoshikazu Nemoto, Hiroyuki Terawaki

**Affiliations:** 1 Department of Internal Medicine, Nephrology, Teikyo University Chiba Medical Center, 3426-3 Anesaki, Ichihara, Chiba Japan; 2 Department of Community Medicine, Teikyo University Chiba Medical Center, Ichihara, Japan; 3 Biomolecular Logic Research Laboratory, Tokyo, Japan

**Keywords:** Chronic kidney disease, Kidney dysfunction, Health check-up, Uric acid, CKD-EPI

## Abstract

**Background:**

The effect of low serum uric acid (sUA) levels on kidney function is unclear. This study aimed to clarify the relationship between low sUA levels and the rapid decline in kidney function.

**Methods:**

We examined the relationship between sUA levels and kidney function decline in health check-up examinees. A total of 10,547 participants were enrolled using data from the Yuport Medical Checkup Center Study between 1998 and 2002 for baseline and data from 2002 to 2006 as the follow-up period in Japan. According to sUA level (mg/dL), we classified the participants into the following six groups: (1) 2.0–2.9 (*n* = 247), (2) 3.0–3.9 (*n* = 1457), (3) 4.0–4.9 (*n* = 2883), (4) 5.0–5.9 (*n* = 2899), (5) 6.0–6.9 (*n* = 2010), and (6) 7.0–7.9 (*n* = 1,051). The relationship between sUA level and rapid decline in estimated glomerular filtration rate (ΔeGFR ≥ 3 mL/min/1.73 m^2^/year) was examined using a logistic regression model.

**Results:**

During study period (5.4 ± 1.6 years), the incidence of rapid eGFR decline for the respective sUA groups (2.0–2.9, 3.0–3.9, 4.0–4.9, 5.0–5.9, 6.0–6.9, 7.0–7.9) were as follows: 4.5%, 4.0%, 2.4%, 3.3%, 3.1%, 3.4%. The crude and adjusted odds ratios (OR) for rapid eGFR decline were significantly higher in the 2.0–2.9 (OR:1.93 and 1.86) and 3.0–3.9 (OR:1.72 and 1.73) groups than in the 4.0–4.9 groups (reference). Stratified analysis of age differences revealed that the detrimental effect of low sUA was not evident in older adults (age ≥ 65 years).

**Conclusion:**

A lower normal sUA level is related to an increased risk for a rapid decline in kidney function.

**Supplementary Information:**

The online version contains supplementary material available at 10.1007/s10157-023-02318-0.

## Introduction

Chronic kidney disease (CKD) is a worldwide clinical issue, because it is not only potential end-stage kidney disease, but it is also an independent risk factor for death and/or cardiocerebrovascular events in general populations. [1–3]. CKD prevention is a critical target for investigation from a public health point of view. CKD is primarily caused by non-communicable, lifestyle-related diseases, such as diabetes or hypertension. As CKD is asymptomatic in the early stages, early detection of potential CKD risk factor(s) through annual checkups is a necessity.

Among potential CKD risk factors, excess serum uric acid (sUA) level, or hyperuricemia, has recently emerged as a risk factor [4–7]. Hyperuricemia causes not only gouty arthritis but also gout kidney—kidney dysfunction due to the deposition of uric acid-sodium (MSU) on the kidney interstitium [8, 9].

On the other hand, uric acid could exhibit beneficial aspects through antioxidative properties [10]. We formerly reported that slightly lower level which is within normal range (≤ 4.6 mg/dL) of serum albumin related to rapid decline in kidney function [11]. The reason of such relationship is unclear, however, antioxidative property of serum albumin might contribute to renoprotection, for serum albumin is the most abundant and thus important antioxidative substance of the extracellular space [12]. Therefore, the lack of sUA could have a detrimental effect on kidney function through different pathophysiology from that of the excess of sUA or hyperuricemia. However, previous studies on this are generally insufficient and diverse [13–16].

The present observational study examined CKD occurrence in participants with low but within the normal range of sUA levels using the large sample size (*n* = 10,547) health follow-up cohort data.

## Methods and subjects

### *Study design and participants (Fig. **1**)*

**Fig. 1:**
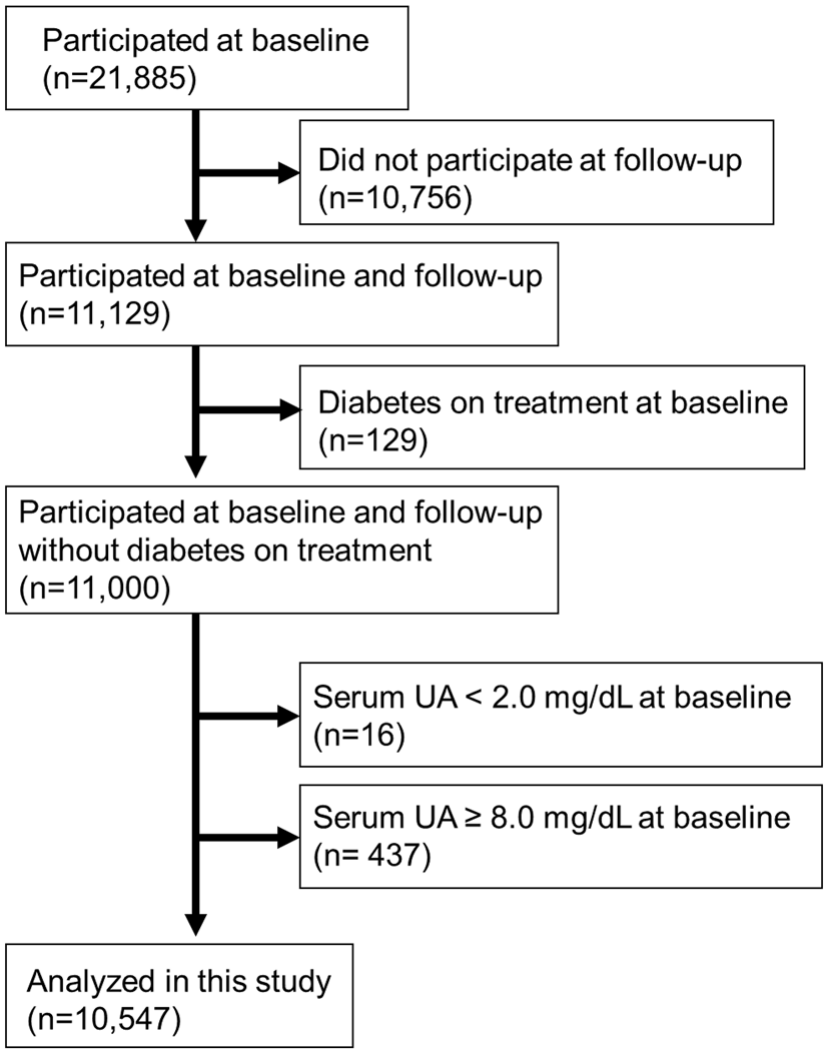
Flowchart of the study population

This was a retrospective cohort study, that used data from health check-up examinees acquired from the health screening program conducted by the Yuport Medical Checkup Center in Tokyo. For the current study, we set a 4-year baseline period between April 1998 and March 2002, and a 4-year follow-up period between April 2002 and March 2006. During the baseline period, 21,885 individuals underwent checkups at least once. Only the first checkup was used as the baseline data for participants who underwent multiple checkups during the baseline period. Of the 11,129 patients who had been examined during both the baseline and follow-up periods, 129 diabetic participants on treatment at baseline were excluded to avoid their potential influence of diabetic kidney disease. Of the remaining 11,000 individuals, participants with hypouricemia (sUA < 2.0 mg/dL; *n* = 16) and hyperuricemia requiring medical treatment (sUA ≥ 8.0 mg/dL; *n* = 437) were also excluded. Accordingly, 10,547 participants were enrolled in this study.

In accordance with the Private Information Protection Law, information that might identify participants was kept private by the center. Informed consent was obtained at every checkup for anonymous participation in epidemiological research.

## Measurements

All checkup procedures, including blood tests, were performed in the same manner at both the baseline and follow-up visits. Blood samples were obtained after overnight fasting and analyses were performed in the center’s laboratory. Uric acid and creatinine levels were measured using enzymatic methods (reagents supplied by Mitsubishi Kagaku Iatron, Tokyo, Japan). Other hematological and biochemical parameters were measured using standard laboratory techniques. Of note, hemoglobin A1c was expressed as the Japan Diabetes Society (JDS) value, and statistical analysis was performed after translation from the JDS value to the National Glycohemoglobin Standardization Program value according to the translation formula published elsewhere [17]. Body mass index (BMI) was defined as weight divided by height squared (kg/m^2^), and it was calculated from the patient’s measured height and weight. Trained nurses measured blood pressure using a sphygmomanometer.

## The kidney function

Kidney function was shown as an estimated glomerular filtration rate (eGFR) using the CKD Epidemiology Collaboration (CKD-EPI) modified for the Japanese population [18]. The CKD-EPI equation, which was estimated by the coefficient-modified equation, was more closely related to the CVD incidence than that estimated by the Japanese GFR equation [19]. The CKD-EPI equation for the Japanese population is as follows:

mCKDEPI-eGFR (mL/min/1.73 m^2^) = 141 × min (Cr/κ, 1)^α^ × max (Cr/κ, 1)^−1.209^ × 0.993^Age^ × 1.018 (if female) × 0.813 (Japanese coefficient), κ:0.7 in female and 0.9 In the male, α was -0.329 in female and -0.411 in male.

Kidney function declines with age. A previous study showed that the mean rate of kidney function decline in people aged 40 years and older was 0.36 mL/min/1.73 m^2^/year [20]. In our study, we defined an “abnormal decline in kidney function” as the difference in the eGFR (ΔeGFR) of ≥ 3 mL/min/1.73 m^2^/year between baseline and follow-up visits for each participant as a cut-off value. According to previous studies, this cut-off value was associated with clinically deleterious outcomes [21, 22]. We also performed a sensitivity analysis that regarded a ΔeGFR of ≥ 5 mL/min 1.73 m^2^/year as an abnormal decline.

### Statistical analysis

Statistical analyses were performed using EZR Version 1.33 (Saitama Medical Center, Jichi Medical University), a graphical user interfaces for R (The R Foundation for Statistical Computing, Vienna, Austria) [23]. EZR is a modified version of the R commander, which is designed to add statistical functions frequently used in biostatistics.

Numeric data were presented as mean ± standard deviation (normally distributed data) or median within the 25th and 75th percentiles (non-normally distributed data) (Appendix 1). Categorical data were expressed as numbers and percentages. *P* < 0.05 (two-tailed) was considered statistically significant.

First, as baseline characteristics, we compared those who met the criteria for an abnormal decline in kidney function with those who did not. Second, we classified participants into six groups (2.0–2.9, 3.0–3.9, 4.0–4.9, 5.0–5.9, 6.0–6.9, and 7.0–7.9 mg/dL) according to their sUA levels. The abnormal decline in kidney function and odds ratio (ORs) for each subject group were estimated using the logistic regression model. Multivariable logistic analyses were used to calculate the odds ratio (OR) for an abnormal decline in kidney function after adjusting for age, sex, BMI, systolic blood pressure, eGFR at baseline, hemoglobin level, serum albumin level, serum alanine aminotransferase level (using logarithmic transformed data), serum non-high-density lipoprotein cholesterol level, serum triglyceride level (using logarithmically transformed data), serum C-reactive protein level (≥ 0.3 mg/dL or not), potential diabetes mellitus suspected from baseline data (fasting plasma glucose ≥ 126 mg/dL or hemoglobin A1c ≥ 6.5%, or both), hypertension on treatment, dyslipidemia on treatment, history of stroke, and ischemic heart disease (angina pectoris or myocardial infarction, or both).

## Results

Table 1 presents the baseline characteristics of the participants according to an abnormal decline in kidney function. Of the 10,547 participants, 333 had a rapid eGFR decline during the study period (5.4 ± 1.6 years). The baseline eGFR (mg/min/1.73 m^2^) of those with rapid decline was significantly higher than that of those with a non-rapid decline (84.3 ± 12.8 vs. 83.1 ± 9.97, *p* < 0.05, *t*-test). Of note, irrespective of the baseline exclusion of known diabetic individuals, still 501 (4.8%) potential diabetic subjects were newly identified.

**Table 1:** Participants’ characteristics at baseline according to decline in kidney function

		Total (*n* = 10,547)	Normal eGFR decliner (*n* = 10,214)	Rapid eGFR decliner (*n* = 333)
Age (years)		53.3 ± 11.6	53.3 ± 11.6	52.0 ± 13.4
Sex (male)		5320 (50.4)	5134 (50.3)	186 (55.9)
BMI (kg/m^2)^		22.9 ± 2.99	22.9 ± 3.0	23.1 ± 3.2
Systolic blood pressure* (mmHg)		123.8 ± 17.8	123.7 ± 17.8	125.8 ± 18.5
Diastolic blood pressure* (mmHg)		74.8 ± 11.0	74.7 ± 11.0	77.0 ± 11.3
140/90 mmHg or higher*		1217 (11.5)	1157 (11.3)	60 (18.0)
Hemoglobin (g/dL)		13.9 ± 1.40	13.9 ± 1.39	13.8 ± 1.64
Albumin* (g/dL)		4.51 ± 0.23	4.52 ± 0.23	4.45 ± 0.23
Alanine transferase (IU/L)		18 (14–25)	18 (14–25)	18 (13–25)
Creatinine (mg/dL)		0.71 ± 0.16	0.71 ± 0.16	0.72 ± 0.17
Estimated GFR* (mL/min/1.73 m^2^)		83.1 ± 10.1	83.1 ± 9.97	84.3 ± 12.8
Uric acid (mg/dL)		5.22 ± 1.23	5.22 ± 1.23	5.22 ± 1.31
Total cholesterol (mg/dL)		203.1 ± 34.8	203.1 ± 34.7	201.4 ± 036.7
HDL cholesterol* (mg/dL)		58.9 ± 15.1	59.0 ± 15.2	55.9 ± 14.3
Lower than 40 mg/dL		812 (7.7)	786 (7.7)	26 (7.8)
Non-HDL cholesterol (mg/dL)		144.2 ± 35.1	144.2 ± 35.0	145.5 ± 36.3
170 mg/dL or higher		2390 (22.7)	2309 (22.6)	81 (24.3)
Triglyceride (mg/dL)		96 (69–137)	96 (69–137)	96 (71–142)
150 mg/dL or higher		2126 (20.2)	2054 (20.1)	72 (21.6)
C-reactive protein (mg/dL)		0.1 (0–0.1)	0.1 (0–0.1)	0.1 (0–0.1)
Fasting plasma glucose (mg/dL)		97 ± 16	97 ± 16	99 ± 22
HbA1c, NGSP (%)		5.4 ± 0.7	5.4 ± 0.7	5.4 ± 1.0
Potential diabetes mellitus from baseline data		501 (4.8)	478 (4.7)	23 (6.9)
Dyslipidemia on treatment		59 (0.56)	57 (0.6)	2 (0.6)
Hypertension on treatment*		325 (3.1)	301 (2.9)	24 (7.2)
History of stroke		20 (0.19)	19 (0.2)	1 (0.3)
History of ischemic heart disease*		43 (0.41)	39 (0.4)	4 (1.2)

According to the property of each datum, the data are expressed as mean and standard deviation, median and quartile range, or subject number and percentage

*P < 0.05 between normal decliner and rapid decliner

Table 2 presents the baseline characteristics of the participants according to their sUA levels. The prevalence of males and hypertension on treatment was increased, in sUA elevated group. (*p* < 0.05) in the Cochran-Armitage test. Similarly, BMI, systolic blood pressure, hemoglobin, serum albumin, alanine transferase, creatinine, non-HDL cholesterol, triglyceride, and C-reactive protein levels were also elevated in the group that had an elevation of sUA (*p* < 0.05). These positive findings in trend tests (Cochran-Armitage and Jonckheere-Terpstra tests) suggest the presence of a confounding relationship between each parameter and the sUA level.

**Table 2:** Participants’ characteristics at baseline according to serum uric acid levels

	UA2.0–2.9 (*n* = 247)	UA3.0–3.9 (*n* = 1457)	UA4.0–4.9 (*n* = 2883)	UA5.0–5.9 (*n* = 2899)	UA6.0–6.9 (*n* = 2010)	UA7.0–7.9 (*n* = 1051)
Age* (years)	50.9 ± 10.6	51.5 ± 11.4	53.8 ± 11.4	54.3 ± 11.7	52.9 ± 11.9	52.7 ± 11.6
Sex* (male)	28 (11.3)	210 (14.4)	745 (25.8)	1718 (59.3)	1653 (82.2)	966 (91.9)
BMI* (kg/m^2)^	21.3 ± 2.4	21.5 ± 2.6	22.3 ± 2.9	23.1 ± 2.9	23.8 ± 2.8	24.5 ± 3.0
Systolic blood pressure* (mmHg)	116.4 ± 16.7	118.3 ± 16.5	122.0 ± 17.5	124.4 ± 18.2	127.5 ± 17.5	129.3 ± 16.7
Hemoglobin* (g/dL)	12.6 ± 1.36	12.9 ± 1.35	13.4 ± 1.25	14.1 ± 1.24	14.6 ± 1.1	14.8 ± 1.1
Albumin* (g/dL)	4.42 ± 0.21	4.46 ± 0.21	4.48 ± 0.22	4.52 ± 0.22	4.56 ± 0.24	4.59 ± 0.24
Alanine transferase* (IU/L)	14 (11–18)	15 (12–20)	16 (13–22)	19 (15–26)	23 (16–31)	25 (18–35)
Creatinine* (mg/dL)	0.58 ± 0.10	0.61 ± 0.11	0.65 ± 0.12	0.73 ± 0.14	0.80 ± 0.14	0.85 ± 0.17
Estimated GFR* (mL/min/1.73 m^2^)	88.2 ± 8.0	86.8 ± 8.7	84.1 ± 8.9	82.3 ± 9.8	81.3 ± 10.9	79.7 ± 11.8
Uric acid* (mg/dL)	2.62 ± 0.24	3.55 ± 0.26	4.47 ± 0.28	5.44 ± 0.29	6.41 ± 0.28	7.38 ± 0.28
Total cholesterol (mg/dL)	197.5 ± 35.8	201.4 ± 35.2	204.2 ± 35.1	203.1 ± 34.4	202.3 ± 34.9	205.1 ± 33.7
HDL cholesterol* (mg/dL)	65.0 ± 14.4	64.8 ± 14.7	62.3 ± 15.1	57.7 ± 14.5	54.3 ± 14.0	52.0 ± 13.8
Non-HDL cholesterol* (mg/dL)	132.5 ± 33.9	136.6 ± 34.4	141.9 ± 34.9	145.4 ± 34.3	148.0 ± 35.5	153.1 ± 35.0
Triglyceride* (mg/dL)	73 (55–102)	75 (58–102)	83 (62–118)	100 (73–137)	114 (81–164)	139 (99–197)
C-reactive protein* (mg/dL)	0 (0–0.1)	0 (0–0.1)	0 (0–0.1)	0.1 (0–0.1)	0.1 (0- 0.1)	0.1 (0–0.1)
Potential diabetes mellitus from baseline data	6 (2.4)	43 (3.0)	129 (4.5)	168 (5.8)	103 (5.1)	52 (4.9)
Dyslipidemia on treat	0	5 (0.3)	19 (0.7)	14 (0.5)	14 (0.7)	7 (0.7)
Hypertension on treat*	0	19 (1.3)	77 (2.7)	112 (3.9)	69 (3.4)	48 (4.6)
History of stroke	0	0	5 (0.2)	8 (0.3)	4 (0.2)	3 (0.3)
History of ischemic heart disease	0	1 (0.1)	15 (0.5)	15 (0.5)	5 (0.2)	7 (0.7)

According to the property of each datum, the data are expressed as mean and standard deviation, median and quartile range, or subject number and percentage

*P for trend < 0.05 (Jonckheere-Terpstra test for numerical data, Cochran-Armitage test for category data)

**Fig. 2:**
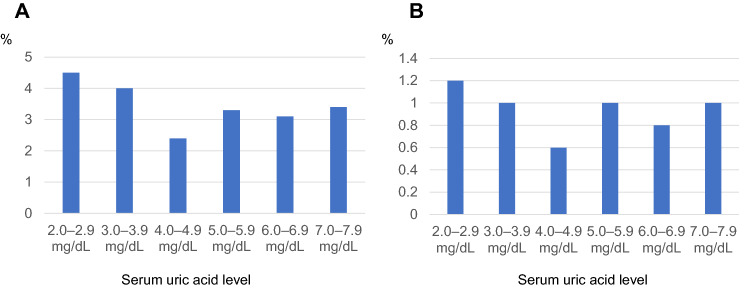
Percentage of participants with significantly reduced kidney function (ΔeGFR of ≥ 3 mL/min 1.73 m^2^/year) by serum uric acid level. The percentage of rapid decliner (ΔeGFR of ≥ 3 mL/min 1.73 m^2^/year) was the lowest with serum uric acid levels of 4.0–4.9 mg/dL (**A**). This tendency was similar in the sensitivity analysis using ΔeGFR of ≥ 5 mL/min 1.73 m^2^/year as the criteria for rapid kidney function decline (**B**)

Figure 2 shows the percentage of participants with significantly reduced kidney function (ΔeGFR ≥ 3 mL/min 1.73 m^2^/year) according to serum uric acid level. The incidence of each subject group was significantly different (*p* < 0.05, in the Chi-square test). Of all the participants’ groups, the 4.0–4.9 group exhibited the lowest incidence (2.4%). Sensitivity analysis also showed the same trend. This finding was similar to that of an analysis which showed incremental increases of 0.5 in sUA levels, the ratios of rapid decline in 4.0–4.4 and 4.5–4.9 were 2.4% and 2.3%, respectively, and those of all the other groups were higher than 2.5% (Appendix 2). Therefore, we regarded the 4.0–4.9 group as the reference group in the following logistic regression analyses.

**Fig. 3:**
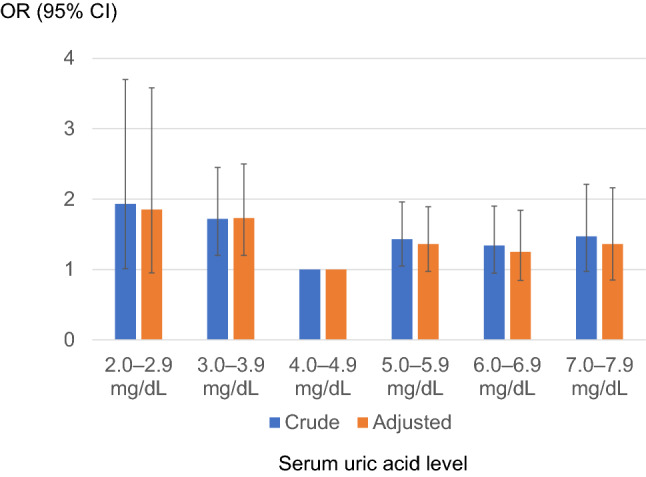
The odds ratio for the rapid decline in kidney function by serum uric acid level classification. Reference level 4.0–4.9 mg/dL. Adjusted for age, sex, BMI, systolic blood pressure, hemoglobin, alanine aminotransferase (logarithmically transformed value), serum albumin, kidney function at baseline, high-density lipoprotein cholesterol, triglyceride (logarithmically transformed value), C-reactive protein (elevated or not), potential diabetes, and other comorbidities (hypertension on treatment, dyslipidemia on treatment, stroke history, and ischemic heart disease history)

Logistic regression analysis was used to calculate the OR for the rapid decline in kidney function according to the sUA level (Fig. 3, Table 3). The OR of rapid eGFR decline was higher in both higher sUA groups and lower normal sUA groups (the so-called J-curve phenomenon). This tendency was also observed after adjusting for multiple confounding factors.

**Table 3:** The odds ratio for rapid decline in kidney function by serum uric acid level classification

sUA class (mg/dL)	OR (95% CI)		*p* value	
	Crude	Adjusted	Crude	Adjusted
2.0–2.9	1.93 (1.01–3.70)	1.85 (0.952–3.58)	0.0477	0.0698
3.0–3.9	1.72 (1.20–2.45)	1.73 (1.20–2.50)	0.0030	0.0031
4.0–4.9	1 (ref.)	1 (ref.)		
5.0–5.9	1.43 (1.05–1.96)	1.36 (0.972–1.89)	0.0249	0.0728
6.0–6.9	1.34 (0.946–1.90)	1.25 (0.845–1.84)	0.0993	0.2650
7.0–7.9	1.47 (0.974–2.21)	1.36 (0.849–2.16)	0.0665	0.2030

A graphic image of this result is shown in Fig. 3. Adjusted for age, sex, BMI, systolic blood pressure, hemoglobin, alanine aminotransferase (log), serum albumin, kidney function at baseline, high-density lipoprotein cholesterol, triglyceride (log), C-reactive protein (elevated or not), potential diabetes and other comorbid status (hypertension, dyslipidemia, history of stroke and ischemic heart disease)

Because 501 participants met the criteria of potential diabetes mellitus at baseline notwithstanding they had not diagnosed as diabetes before (Table 1), we performed logistic regression analysis using data set without potential diabetes at baseline to omit profound effect of early-stage diabetes on eGFR decline. As a result, same trend as analysis including the “potentially diabetic” participants was obtained (Appendix 3).

**Fig. 4:**
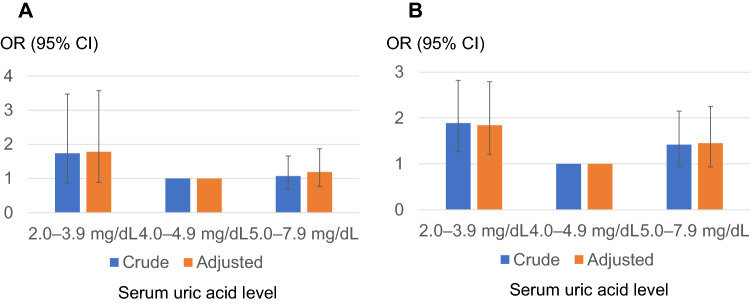
The odds ratio for men (**A**) and women (**B**) regarding the rapid decline in kidney function by serum uric acid level classification. Reference level 4.0–4.9 mg/dL. Adjusted for age, BMI, systolic blood pressure, hemoglobin, alanine aminotransferase (logarithmically transformed value), serum albumin, kidney function at baseline, high-density lipoprotein cholesterol, triglyceride (logarithmically transformed value), C-reactive protein (elevated or not), potential diabetes, and other comorbidities (hypertension, dyslipidemia, stroke, and ischemic heart disease)

**Fig. 5:**
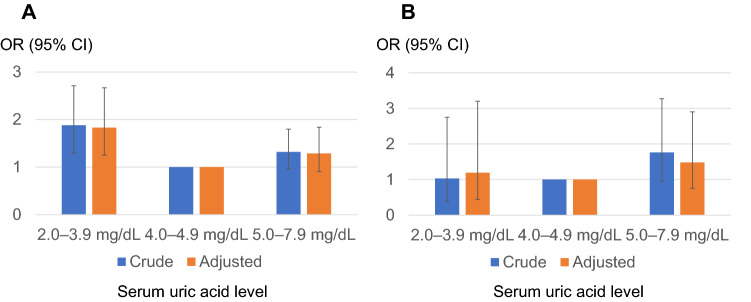
The odds ratio for young and middle-aged adults (**A**) and older adults (**B**) regarding the rapid decline in kidney function by serum uric acid level classification. Reference level 4.0–4.9 mg/dL. Adjusted for sex, BMI, systolic blood pressure, hemoglobin, alanine aminotransferase (logarithmically transformed value), serum albumin, kidney function at baseline, high-density lipoprotein cholesterol, triglyceride (logarithmically transformed value), C-reactive protein (elevated or not), potential diabetes, and other comorbidities (hypertension, dyslipidemia, stroke, and ischemic heart disease)

**Fig. 6:**
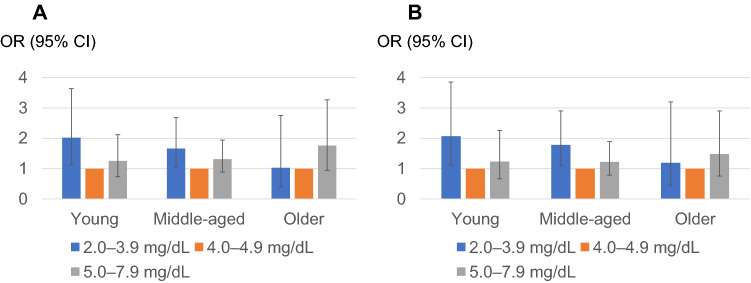
The crude (**A**) and adjusted (**B**) odds ratio for age groups (young, middle-aged, or older adults) regarding the rapid decline in kidney function by serum uric acid level classification. Reference level 4.0–4.9 mg/dL. Adjusted for sex, BMI, systolic blood pressure, hemoglobin, alanine aminotransferase (logarithmically transformed value), serum albumin, kidney function at baseline, high-density lipoprotein cholesterol, triglyceride (logarithmically transformed value), C-reactive protein (elevated or not), potential diabetes, and other comorbidities (hypertension, dyslipidemia, stroke, and ischemic heart disease)

Then, we performed stratified analyses for sex and age. In the stratified analysis after classification on the basis of sex (Fig. 4), a similar trend was observed in both males and females. Likewise, we stratified the analysis regarding age, in which participants were divided into the following two groups: young and middle-aged adults (ages 19–64 years; *n* = 8660) and older adults (aged 65 years and over; *n* = 1887) (Fig. 5). Unlike in young and middle-aged adults, the OR of the lower normal sUA (2.0–3.9) group was not elevated [crude OR 1.03 (95% confidence interval [CI] 0.643–1.72) and adjusted OR was 1.19 (95% CI 0.752–2.01)] in older adults. A similar tendency was observed when participants were divided into the following three groups: young adults (ages 19–44 years; *n* = 2512), middle-aged adults (ages 45–64 years; *n* = 6148), and older adults (aged 65 years and over; *n* = 1887) (Fig. 6). The OR for rapid eGFR decline in the lower normal sUA group (2.0–3.9) in older adults was not elevated in either crude or adjusted analyses.

## Discussion

The aim of this study was to clarify the relationship between lower normal sUA levels and rapid decline in kidney function using health check-up examinee cohort data from a large sample size. As a result, we clarified that lower normal sUA levels (2.0–3.9) were independently associated with rapid eGFR decline. Of course, the pathogenesis of such mild hypouricemia in this study might include profound insufficiency in proximal tubular function. In our analyses, the uric acid level at the lowest risk of rapid eGFR decline was 4.0–4.9 mg/dL.

One of the novelties of our present study is that the contribution of lower normal sUA to rapid decline of eGFR was ensured in a prospective manner. Although recent cohort studies showed the relationship between lower sUA and kidney dysfunction [15, 16], these studies were cross-sectional and thus cause-and-result relationship was unclear.

Regarding the clinical impact, incidence, and pathogenesis of rapid eGFR decline in general, Rifkin et al. evaluated in their cohort of community-dwelling older adults recruited individuals aged ≤ 65 years from Medicare eligibility lists in 4 US communities (Forsyth County, North Carolina; Sacramento County, California; Washington County, Maryland; and Pittsburgh, Pennsylvania) [21, 22]. In their cohort, the clinical impact of rapid eGFR decline was statistically significant relationship with increased risk of cardiovascular and all-cause mortality [21]. The incidence of rapid eGFR decline was 16–25% [21, 22], which was quite higher than that in present study (3.16%), suggesting the racial difference between their cohort and ours. As to the pathogenesis, rapid eGFR decline in their cohort was independently associated with systolic blood pressure (SBP) [22]. Such significant relationship between rapid decline of eGFR and SBP was also confirmed among our present study.

Another novelty of this study is that the profound effect of “metabolic” multiple confounding factors (such as obesity, diabetes, hypertension, dyslipidemia, liver disease, chronic inflammation, and serum albumin level) was minimized through study design (exclusion of participants being treated for diabetes) and statistical correction. In particular, correction of serum albumin level, one of the important predictors of renal prognosis probably through antioxidative property [11] (Appendix 4), has seldom been performed in previous studies.

It was already known in the early 1980s that uric acid has a strong antioxidant effect [24]. Such antioxidant effect of uric acid could contribute to longevity of hominoids—which abolished the activity of uricase (an enzyme that breaks down uric acid to allantoin) 15,400,000 years ago, and thus which sUA level is higher than other primates [25]. The antioxidant activity of uric acid surpasses that of vitamin C [10], and clinically, uric acid has been proposed to have a neuroprotective effect due to its antioxidant effect in the field of the central nervous system, especially in multiple sclerosis, Parkinson’s disease, and acute cerebral infarction [26–29]. In addition, a low level of uric acid causes oxidative stress in endothelial cells, leading to the induction of oxidative apoptosis, expression of adhesion molecules, and reduction of microvessels [30–32].

Animal study regarding the effect of hypouricemia on kidney function is not present, because mammalian species except for Hominidae have uricase—an enzyme that promotes oxidation of uric acid to allantoin—and thus almost all animals (except for ape and human) are “hypouricemic” fundamentally. On the other hand, Cutler evaluated the relationship between serum and brain urate level (indexed for specific metabolic rate) and the maximum lifespan potential of various animals (22 primate and 17 non-primate mammalian species) and found the positive correlation between them [33]. This finding from inter-species comparison suggests organoprotective effect of uric acid, and accordingly, individual with lower sUA might be vulnerable to organic free radicals constantly caused by living activity.

However, high sUA levels also exhibit detrimental effects on kidney function, as is widely acknowledged [4–7]. Uric acid is involved in promoting oxidation within cells. This is because reactive oxygen species (superoxide and hydrogen peroxide) are produced in the process of uric acid synthesis by xanthine oxidase (XO) (reactive oxygen species producing-type isoform of uric acid synthase xanthine oxidoreductase (XOR)) leading to vascular endothelial damage and organ damage [34]. This might be the reason why high uric acid levels increase the risk of various complications including hypertension, diabetes, cerebrovascular and cardiovascular disease, metabolic syndrome, and CKD, even if the uric acid level is 7.0 mg/dL or less which could not cause MSU deposition in organs. We previously ensured among CKD patients that the oxidized serum albumin ratio (a marker of oxidative stress) showed an independent positive correlation with serum UA level [35] and XO / XOR ratio [36]. Furthermore, Uedono et al. clarified in a study of kidney transplant donor candidates that even if the serum uric acid levels were within the normal range, both mildly low (< 3.5 mg/dL) or high (> 6.0 mg/dL) sUA levels were associated with increased afferent arteriole resistance and decreased blood flow and glomerular filtration rate [37]. To summarize, sUA levels that are "not too low nor too high" may provide the greatest benefit for kidney function protection. Further clinical investigations using kidney biopsy specimens might offer more precise findings regarding the pathophysiology.

It is widely acknowledged that sUA levels in men are generally higher than those in women. However, the statistical relationship between sUA and the clinical characteristics of metabolic syndrome is stronger in women than in men [38]. Thus, we performed a stratified analysis regarding sex, considering the differences in sUA conditions between females and males. As a result, we could not find evident differences in the relationship between sUA levels and rapid eGFR decline, suggesting that such a relationship did not differ between the sexes.

In the present study, regarding the rapid decrease of kidney function, the detrimental effect of low UA was evident not in older adults (aged 65 years and over) but in young and middle-aged adults (ages 19–64 years)—“under 65” generation (Fig. 6). To the best of our knowledge, such a clear “generation gap” regarding the effect of lower sUA level on kidney function has not been pointed out in any previous reports. The need for social and physiological activity is higher in the “under 65” generation than in the “over 65” generation, for most of the “over 65” person retires from a social role such as working or child-rearing. In “under 65” generation, the demand for uric acid to combat oxidative stress caused by high activity [39] could be more earnest than in the “over 65” generation.

This study had some limitations. First, this study targeted health check-up examinees—such a population might have high health literacy and might be at a lower risk of developing the disease when compared to the general population. Second, information regarding regular medication is not known in this cohort, although it was confirmed that participants had not taken oral urate-lowering drugs at baseline, we do not know whether urate-lowering drugs were started after that. Third, information regarding urinary findings is lacking in this cohort, which impedes the evaluation of albuminuria, the major influencer of future kidney damage. Finally, the present cohort consisted of Asian (mainly Japanese) individuals. Thus, future prospective studies addressing these limitations are warranted.

In conclusion, our study showed that a lower normal sUA level is independently related to the risk of a rapid decline in kidney function, especially in young and middle-aged adults.

## Supplementary Information

Below is the link to the electronic supplementary material.

Supplementary file1 (PDF 343 KB)

## Data Availability

The dataset used in this study is under the control of the Data Management Committee of the Yuport Medical Checkup Center (YMCC) study and cannot be shared publicly.  However, when the researcher needs to use the data for the individual patient level meta-analysis or the validation study between another independent cohort, the data set will be available after the review of the Data Management Committee of the YMCC study. The amended protocol will need to be approved by the ethical committee of Teikyo University School of Medicine. Send a request to Hiroyuki Terawaki, M.D., Ph.D., Teikyo University Chiba Medical Center, terawaki@med.teikyo-u.ac.jp.
