# Supplementary material for: Lower range of serum uric acid level increases risk of rapid decline of kidney function in young and middle-aged adults: the Yuport Medical Checkup Center Study
Source: Clin Exp Nephrol. 2023 Feb 11;27(5):435–44. doi: 10.1007/s10157-023-02318-0 (PMC10104940; doi:10.1007/s10157-023-02318-0)
Supplement: Supplementary file 1 — Supplementary file1 (PDF 343 KB) [file 10157_2023_2318_MOESM1_ESM.pdf]

Age

One-sample Kolmogorov-Smirnov test

D = 0.054824, p-value < 2.2e-16
alternative hypothesis: two-sided

Skewness -0.246
Kurtosis -0.527

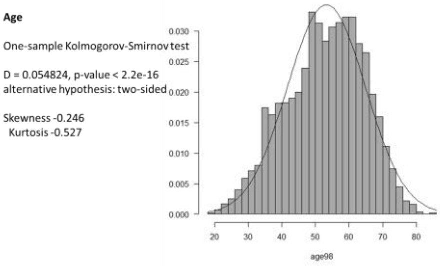

Serum albumin

One-sample Kolmogorov-Smirnov test

D = 0.091036, p-value < 2.2e-16
alternative hypothesis: two-sided

Skewness 0.0706
Kurtosis 0.1860

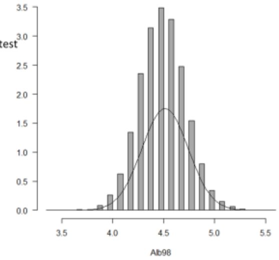

Alanine transferase

One-sample Kolmogorov-Smirnov test

D = 0.19163, p-value < 2.2e-16
alternative hypothesis: two-sided

Skewness 5.37
Kurtosis 61.50

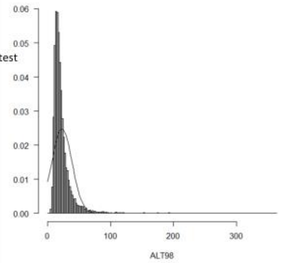

Alanine transferase (log)

One-sample Kolmogorov-Smirnov test

D = 0.078429, p-value < 2.2e-16
alternative hypothesis: two-sided

Skewness 0.83
Kurtosis 1.59

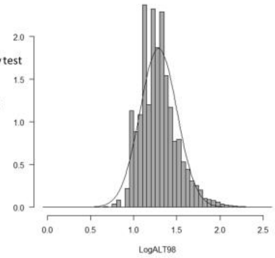

BMI

One-sample Kolmogorov-Smirnov test

D = 0.028367, p-value = 0.00000008507
alternative hypothesis: two-sided

Skewness 0.541
Kurtosis 0.930

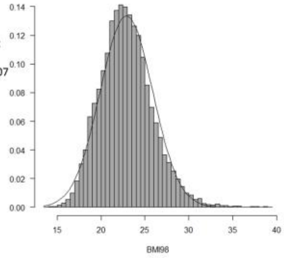

CRP

One-sample Kolmogorov-Smirnov test

D = 0.36417, p-value < 2.2e-16
alternative hypothesis: two-sided

Skewness 12.3
Kurtosis 228.0

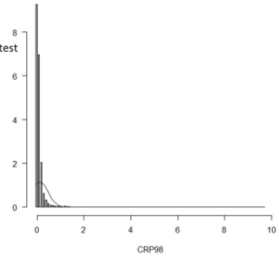

CRP (root)

One-sample Kolmogorov-Smirnov test

D = 0.26404, p-value < 2.2e-16
alternative hypothesis: two-sided

Skewness 2.24
Kurtosis 12.00

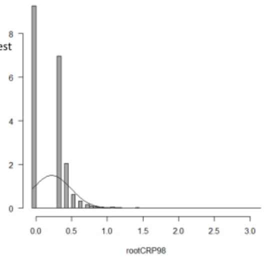

Yearly eGFR decline

One-sample Kolmogorov-Smirnov test

D = 0.21501, p-value < 2.2e-16
alternative hypothesis: two-sided

Skewness -1.34
Kurtosis 24.60

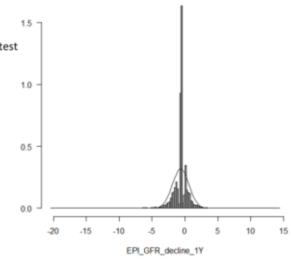

Estimated GFR

One-sample Kolmogorov-Smirnov test

D = 0.037143, p-value = 4.594e-13
alternative hypothesis: two-sided

Skewness -0.458
Kurtosis 1.420

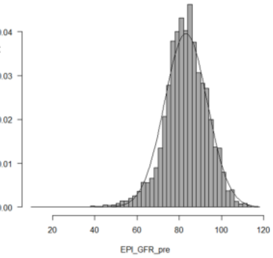

Hemoglobin

One-sample Kolmogorov-Smirnov test

D = 0.031671, p-value = 0.000000001295
alternative hypothesis: two-sided

Skewness -0.370
Kurtosis 0.865

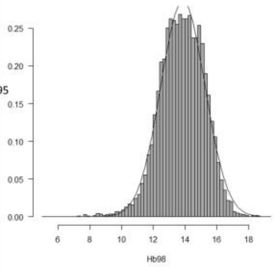

HDL-cho

One-sample Kolmogorov-Smirnov test

D = 0.056855, p-value < 2.2e-16
alternative hypothesis: two-sided

Skewness 0.633
Kurtosis 0.490

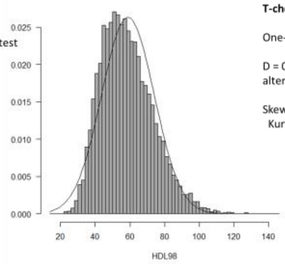

T-cho

One-sample Kolmogorov-Smirnov test

D = 0.028745, p-value = 0.00000005388
alternative hypothesis: two-sided

Skewness 0.352
Kurtosis 0.448

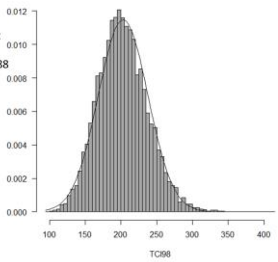

Non-HDL cholesterol

One-sample Kolmogorov-Smirnov test

D = 0.023661, p-value = 0.00001488
alternative hypothesis: two-sided

Skewness 0.369
Kurtosis 0.395

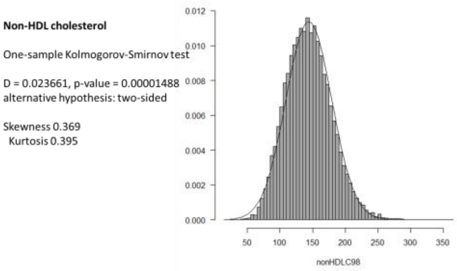

TG

One-sample Kolmogorov-Smirnov test

D = 0.14202, p-value < 2.2e-16
alternative hypothesis: two-sided

Skewness 5.14
Kurtosis 79.90

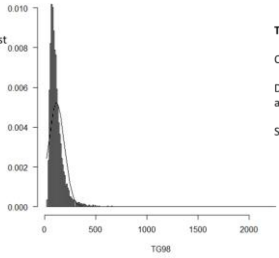

TG (log)

One-sample Kolmogorov-Smirnov test

D = 0.031879, p-value = 9.8e-10
alternative hypothesis: two-sided

Skewness 0.458
Kurtosis 0.497

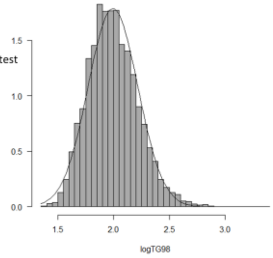

Appendix 1

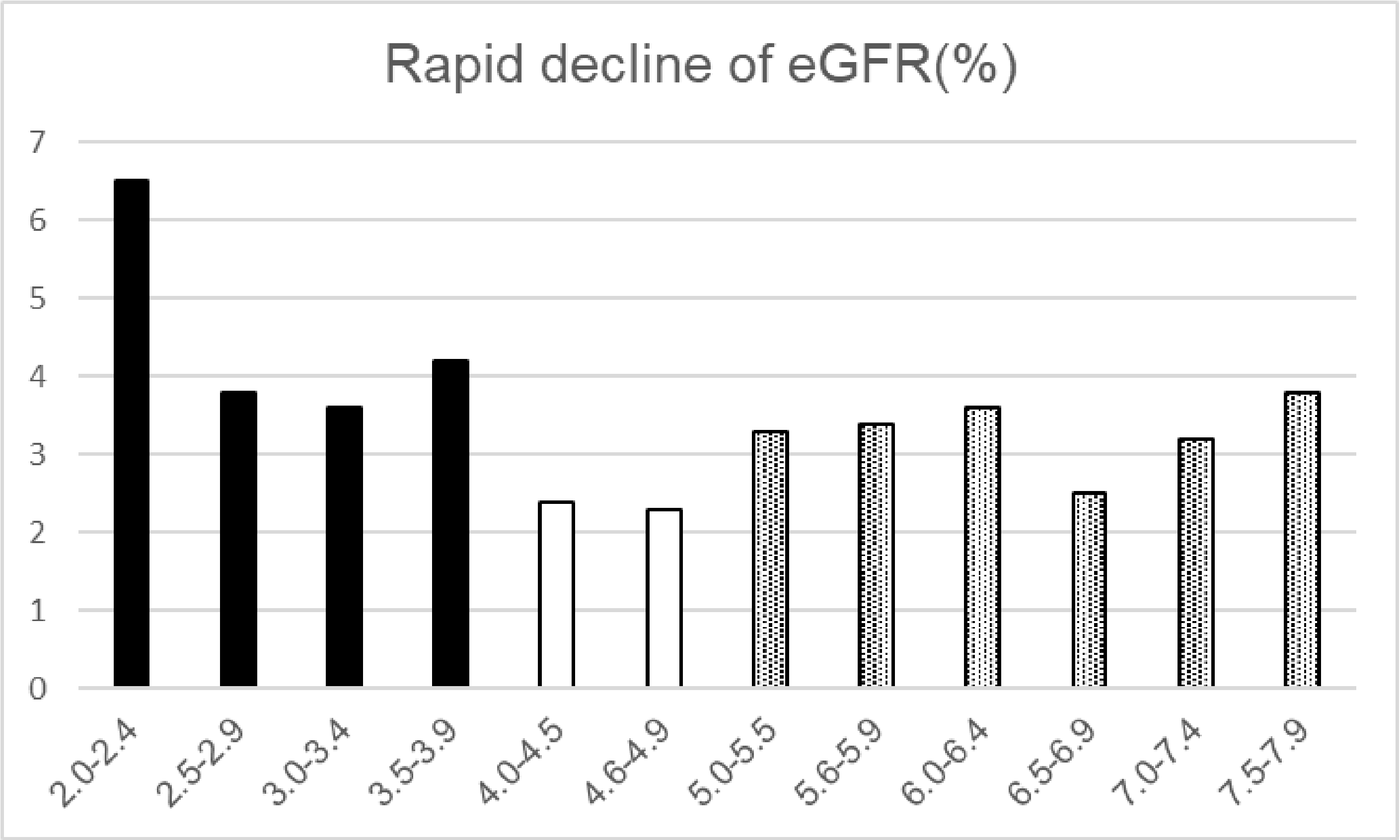
Rapid decline of eGFR(%)
7
6
5
4
3
2
1
0
2.0-2.4
2.5-2.9
3.0-3.4
3.5-3.9
4.0-4.5
4.6-4.9
5.0-5.5
5.6-5.9
6.0-6.4
6.5-6.9
7.0-7.4
7.5-7.9

Appendix 2

	Odds ratio	95% CI	P-value
(Intercept)*	0.023	0.018 - 0.030	1.96×10^{-189}
Serum UA level (mg/dL)			
2.0 to 2.9*	2.08	1.08 – 4.00	2.88×10^{-2}
3.0 to 3.9*	1.79	1.24 – 2.58	1.87×10^{-3}
4.0 to 4.9	1	(ref.)	(ref.)
5.0 to 5.9*	1.50	1.08 – 2.08	1.57×10^{-2}
6.0 to 6.9*	1.31	0.91 – 1.89	1.44×10^{-1}
7.0 to 7.9*	1.53	1.00 – 2.34	4.98×10^{-2}

*; P<0.05

Appendix 3

	Odds ratio	95% CI	P-value
(Intercept)	11.0	0.424 – 285	1.49×10^{-1}
Serum UA level (mg/dL)			
2.0 to 2.9*	1.89	0.978 – 3.65	5.84×10^{-2}
3.0 to 3.9*	1.75	1.22 – 2.52	2.41×10^{-3}
4.0 to 4.9	1	(ref.)	(ref.)
5.0 to 5.9	1.37	0.982 – 1.91	6.39×10^{-2}
6.0 to 6.9	1.23	0.833 – 1.81	2.99×10^{-1}
7.0 to 7.9	1.34	0.840 – 2.13	1.61×10^{-1}
Age*	0.982	0.987 – 0.997	1.98×10^{-2}
Male gender	1.30	0.970 – 1.71	6.19×10^{-2}
BMI (kg/m^2)	0.998	0.958 – 1.04	9.23×10^{-1}
Albumin (g/dL)*	0.171	0.102 – 0.288	3.00×10^{-12}
Estimated GFR (mL/min/1.73 m^2)	1.01	0.992 – 1.03	3.18×10^{-1}
Sysytolic BP (mmHg)*	1.01	1.00 – 1.02	9.52×10^{-4}
Non-HDL cholesterol >=170 mg/dL	1.00	1.00 – 1.01	7.96×10^{-2}
Triglyceride >=150 mg/dL	1.00	0.999 – 1.00	5.31×10^{-1}
Potential diabetes*	1.57	0.997 – 2.46	5.15×10^{-2}

*; P<0.05

Appendix 4
